# MeCP2 and MBD2 expression in human neoplastic and non-neoplastic breast tissue and its association with oestrogen receptor status

**DOI:** 10.1038/sj.bjc.6601392

**Published:** 2003-11-11

**Authors:** H M Müller, H Fiegl, G Goebel, M M Hubalek, A Widschwendter, E Müller-Holzner, C Marth, M Widschwendter

**Affiliations:** 1 Department of Obstetrics and Gynecology, University Hospital of Innsbruck, Anichstrasse 35, 6020 Innsbruck, Austria; 2 Department of Biostatistics and Documentation, University of Innsbruck, Schöpfstrasse 41/1, 6020 Innsbruck, Austria

**Keywords:** MeCP2, MBD2, oestrogen receptor, breast cancer, methyl-CpG-binding protein

## Abstract

This study analysed mRNA expression of two members of the methyl-CpG-binding protein family – MeCP2 and MBD2 – in human non-neoplastic (*n*=11) and neoplastic (*n*=57) breast tissue specimens using a quantitative real-time PCR method. We observed higher expression levels of MeCP2 mRNA in neoplastic tissues than in non-neoplastic tissues (*P*=0.001), whereas no significant differences for MBD2 were detected. When studying the relations between the most important clinicopathologic features of breast cancer and the mRNA expression level of both MBDs, we found that oestrogen receptor (OR)-positive breast cancer specimens contained higher levels of MeCP2 mRNA than did OR-negative cancers (*P*=0.005). Furthermore, we observed statistically significantly higher levels of MeCP2 in non-neoplastic tissues expressing high levels of OR as compared to those expressing low levels (*P*=0.017). Finally, using a linear regression model, we identified a statistically significant association between OR expression and MeCP2 mRNA expression in neoplastic and non-neoplastic breast tissue specimens (*P*=0.003). In conclusion, we were able to demonstrate for the first time that there exists a strong association between OR status and MeCP2 mRNA expression. Furthermore, we speculate that MeCP2, regulated by OR, plays a key role in the differentiation processes in human breast tissues.

Changes in the DNA methylation status are one of the most common molecular alterations in human neoplasia. Hypermethylation of CpG islands, especially when located in the promoter region, generally causes a loss of expression on the part of genes possessing this structure at their 5′ end (Jones and Baylin, 2002; Laird, 2003; Müller and Widschwendter, 2003). The so-called methyl-CpG-binding proteins (MBDs) are important constituents of the DNA methylation machinery, since they are directly involved in mediating the epigenetic signal (Bird and Wolffe, 1999). The five MBD proteins known to date share a conserved methyl-CpG-binding domain (MBD), which is 70–75 amino acids in length and exhibits 50–70% similarity between the proteins. Four of them are associated with the transcriptional repression of methylated templates in vertebrates and bind methylated DNA (Wade, 2001). MeCP2 is the best studied member of the MBD family: MeCP2 is an abundant chromosome-binding protein that selectively binds 5-methyl cytosine residues in symmetrically positioned CpG dinucleotides in the mammalian genome (Lewis *et al*, 1992). MeCP2 contains two functional domains, an 85-amino-acid MBD essential for its binding to 5-methylcytosine (Nan *et al*, 1993), and a 104-amino-acid transcriptional repression domain (TRD) that interacts with histone deacetylase and the transcriptional corepressor Sin3A.

In addition to MeCP2, MBD2 is another family member already studied in human neoplasia (Kanai *et al*, 1999; Billard *et al*, 2002). MeCP2 and MBD2 differ in several ways with regard to their binding capacities: MeCP2 can recognise a single symmetrically methylated CpG (Meehan *et al*, 1992). MBD2 can bind, *in vitro*, to DNA sequences containing a few methylated CpGs (Hendrich and Bird, 1998); however, the MeCP1 complex containing this protein binds only to densely methylated DNA (Meehan *et al*, 1989). Invalidation of MeCP2 in mice indicates that MeCP1 cannot compensate for the absence of MeCP2 (Chen *et al*, 2001; Guy *et al*, 2001), and, conversely, invalidation of MBD2 does not prompt the neurological disorders observed in MeCP2-null mice mutants (Hendrich *et al*, 2001). The absence of compensation between these proteins suggests that these two MBDs play specific roles in cellular physiology. Although the expression patterns of the MBDs are not yet fully determined, it has been shown in mouse and rat that these genes are expressed at various levels depending on the cell type and the differentiation state (Tate *et al*, 1996). In adult rodent somatic tissues, MeCP2 expression level varies between tissues. For example, the brain has the highest level and the testis the lowest (Meehan *et al*, 1992).

In addition to the loss of MBD2 and MeCP2 in human cancers (Müller-Tidow *et al*, 2001), it has been shown in colon cancer cell lines that MBD2 is associated with methylated promoters of silent p14/p16 gene, and this methylation-dependent association seems to be responsible for their silencing (Magdinier and Wolffe, 2001). Only one study concentrated on the expression levels of MBD2 and MeCP2 in human breast cancer, indicating a loss of MBD2 expression in breast cancer specimens, but no alteration of the expression level of MeCP2 (Billard *et al*, 2002). Moreover, an upregulation of MBD2 and MeCP2 has been described during differentiation in foetal breast tissue (Billard *et al*, 2002).

Breast cancer is the most common malignancy among women in most Western countries. Oestrogen receptor *α* (OR-*α*) is a ligand-activated nuclear receptor that regulates the transcription of oestrogen-responsive genes in diverse target cells (Govind and Thampan, 2001). Oestrogen receptor alpha expression and activation through oestrogens have long been linked to mammary carcinogenesis, breast tumour progression and outcome of breast cancer patients (Fishman *et al*, 1995). Approximately two-third of breast cancers express the OR gene and synthesise OR protein. These tumours tend to be more differentiated and are often responsive to endocrine therapy. One-third of all breast cancers lack the OR and these tumours are generally associated with poorer histological differentiation, higher growth fraction and somewhat worse clinical outcome than OR-positive breast cancers (McGuire, 1978). Therefore, OR can be designated as a surrogate marker for better differentiation of breast cancer tissue.

In order to gain a better insight into the involvement of MBDs in breast cancer, we investigated the expression levels of MeCP2 and MBD2 in human non-neoplastic and neoplastic breast tissue in general using quantitative real-time PCR. We also addressed the question of whether any relations exist between the most important clinicopathologic features of breast cancer and the expression levels of MeCP2 and MBD2.

We were able to demonstrate that there exists a strong association between OR status and the MeCP2 mRNA expression. We speculate that MeCP2, which is one of the most important mediators of the epigenetic signal, is regulated by OR and therefore plays a key role in the differentiation processes in human breast tissues.

## MATERIALS AND METHODS

### Tissue samples

Breast cancer specimens (*n*=57) and non-neoplastic breast tissue (*n*=11) were obtained immediately after resection of the breast or lumpectomy at the Department of Obstetrics and Gynecology, University Hospital of Innsbruck. Specimens were brought to our pathologist, and a part of the tissue was placed into liquid nitrogen and stored at −70°C until lyophilisation.

Of the 11 non-neoplastic specimens, five (46%) were diagnosed as fibroadenomas, two (18%) as papillomatosis and four (36%) as fibrocystic disease (grades I–III). The mean age at diagnosis of this group of patients was 43.7 years. Of the 57 breast cancer patients, 12 (21%) patients were diagnosed with pT I, 41 (72%) with pT II, two (3.5%) with pT III and two (3.5%) with pT IV of the disease. A total of 25 (44%) patients were nodal negative and 32 (56%) patients nodal positive. These included 40 (70%) invasive ductal carcinomas, eight (14%) lobular carcinomas and nine (16%) carcinomas otherwise differentiated (medullary, mucinous, papillary and tubular carcinomas). Oestrogen receptor and progesterone receptor (PR) status were identified immunohistochemically and/or biochemically. In all, 18 (32%) cancer specimens were hormone receptor negative, while 39 (68%) were hormone receptor positive (defined as OR and/or PR positive): 18 (32%) were OR and PR negative, two (3%) were OR negative and PR positive, four (7%) were OR positive and PR negative and 33 (58%) were OR and PR positive. The mean age at diagnosis of this group of patients was 62.9 years.

### RNA extraction and RT reaction

Total cellular RNA was extracted from the tumour specimens using the acid guanidium thiocyanate–phenol–chloroform method as recently described (Widschwendter *et al*, 2000). Integrity was evaluated by assessing the 18S- and 28S-ribosomal RNA bands in 2% ethidium bromide-stained agarose gel. RNA concentration was measured by spectrophotometric analysis. For PR mRNA expression analysis, total RNA was treated with deoxyribonuclease to remove any contaminating genomic DNA.

DNase treatment of RNA was performed in a final volume of 30 *μ*l containing 1 × RT-Buffer (50 mM Tris-HCl, pH 8.3, 75 mM KCl, 5 mM MgCl_2_), 10 U of rRNasin® RNase Inhibitor (Promega, Madison, WI, USA), 20 U of RNase-free DNase (Boehringer Mannheim, 7767852, Mannheim, Germany) and 4 *μ*g of isolated RNA. After the addition of DNase, the mix was incubated for 60 min at 37°C and inactivated at 95°C for 10 min.

Reverse transcription of RNA was performed in a final volume of 20 *μ*l containing 1 × RT-Buffer (50 mM Tris-HCl, pH 8.3, 75 mM KCl, 5 mM MgCl_2_), 40 U of rRNasin® RNase Inhibitor (Promega, Madison, WI, USA), 10 mM dithiothreitol, 200 U of M-MLV Reverse Transcriptase (Gibco BRL, Gaithersburg, MD, USA), 5 *μ*M random hexamers (Applied Biosystems, Foster City, CA, USA) and 400 ng of total RNA. The samples were first incubated at 65°C for 5 min and then quick-chilled on ice. After adding the M-MLV enzyme, the samples were incubated at 25°C for 10 min and at 37°C for 50 min, followed by a period of 15 min at 70°C to inactivate the reverse transcriptase enzyme.

### Primers and probes

Primers and probes for MBD-2 and MeCP-2 were used according to a recently published study (Müller *et al*, 2000), primers and probes for OR-*α* according to Iwao *et al* (2000) and for the TATA box-binding protein (TBP; a component of the DNA-binding protein complex TFIID as an endogenous RNA control) according to Bieche *et al* (2001). Primers and probes for PR were determined with the assistance of the computer program Primer Express (Applied Biosystems, Foster City, CA, USA). BLASTN searches were conducted to confirm the total gene specificity of the nucleotide sequences chosen for the primers and probes. To prevent amplification of contaminating genomic DNA, the probe was placed at the junction between two exons. *PR* (forward-primer): 5′-TCA ACT TAC AAA ACT TCT TGA TAA CTT GC-3′; *PR* (reverse-primer): 5′-GCC CGG GAC TGG ATA AAT G-3′; *PR* (probe): 5′ FAM-TGA TCT TGT CAA ACA ACT TCA TCT GTA CTG CTT GA-3′TAM.

### Real-time PCR amplification

PCR reactions were performed using an ABI Prism 7700 Detection System (Applied Biosystems, Foster City, CA, USA) with a total volume of 25 *μ*l reaction mixture containing 5 *μ*l of each appropriately diluted RT sample (standard curve points and patient samples), 12.5 *μ*l TaqMan Universal PCR Master Mix (Applied Biosystems, Foster City, CA, USA), 900 nM of each primer and 250 nM of the probe. The thermal cycling conditions comprised an initial incubation at 50°C for 2 min, a denaturing step at 95°C for 10 min and 40 cycles at 95°C for 15 s and at 65°C for 1 min. Each experiment included a standard curve with five cDNA concentrations, a control sample (HS578T cell-line for MBD-2 and MeCP-2 assays, T47D cell-line for OR-*α* and MCF-7 for PR), 25 patients and no template control. The standard curves were generated using serially diluted solutions of standard cDNA derived from the HTB-77 carcinoma cell line for MBD-2 and MeCP-2 assays, from MCF-7 for OR-*α* and from T47D for PR assays. Real-time PCR assays were conducted in triplicate for each sample, and the mean value was used for calculation.

### Statistical analysis

To determine the transcription level, the amounts of the target and endogenous reference were determined from the standard curve. For value normalisation, the target amount was divided by the endogenous reference. In order to verify the reproducibility of each assay, the normalised control value was determined. Only experiments in which the control value was ±2 s.d. were accepted.

For determination of univariate relationships between groups, the Mann–Whitney *U*-test or (for clinicopathologic features with more than two stages) the Kruskal–Wallis test was used. Multiple testing situations were addressed using Bonferroni correction. The Spearman correlation analysis was used to specify relationships between MBD2 and MeCP2, as well as between OR and PR. The results are expressed as *R* values ranking from −1 (inverse correlation) to +1 (positive correlation), with 0 indicating no correlation. Multiple effects and interactions of predictors were analysed using a linear regression model for continuous dependent variables, and a logistic regression model for dichotomous outcome variables. The level of statistical significance was set at *P*⩽0.05. All analyses were performed using SPSS statistical software. Figures are shown without outliers.

## RESULTS

### mRNA expression of MeCP2 and MBD2 in non-neoplastic and neoplastic breast tissue specimens

**Figure 1:**
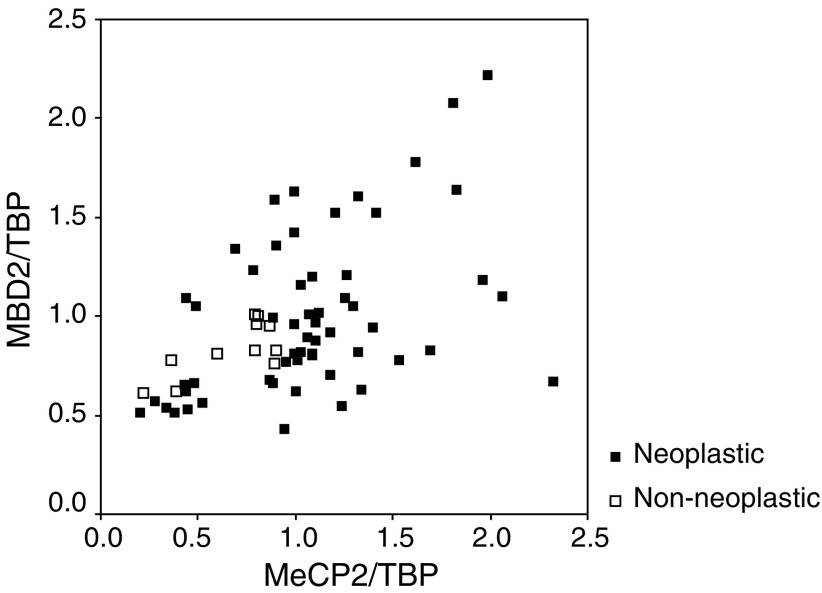
Expression of MeCP2 mRNA in non-neoplastic (*n*=11) and neoplastic (*n*=57) breast tissue specimens. Expression levels were analysed using a quantitative real-time PCR method, as described in Materials and Methods. Expression levels were standardised using TBP expression. A strong correlation between the expression levels of MeCP2 and MBD2 mRNA was seen in both tissue types.

**Figure 2:**
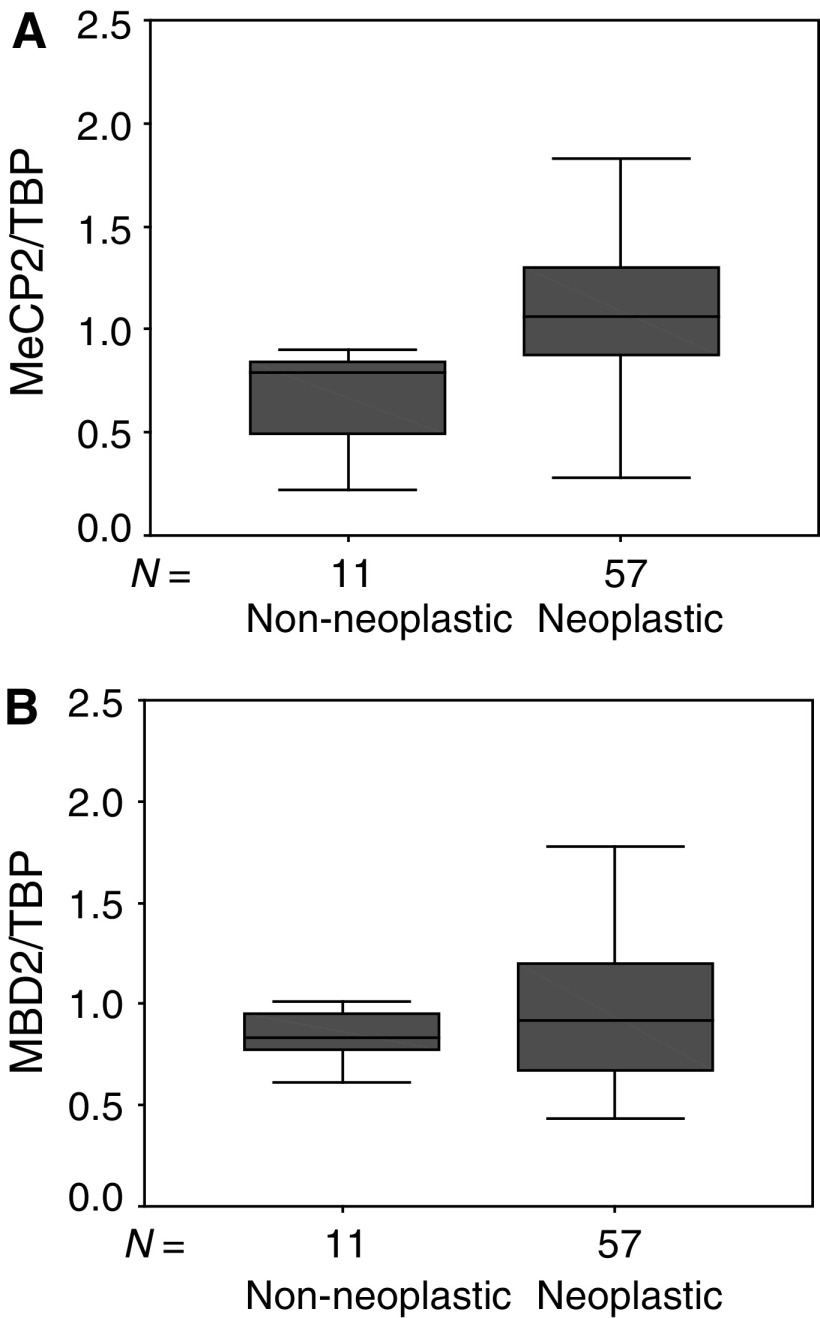
(**A**) Expression of MeCP2 mRNA in non-neoplastic (*n*=11) and neoplastic (*n*=57) breast tissue specimens. Expression levels were analysed using a quantitative real-time PCR method, as described in Materials and Methods. Expression levels were standardised using TBP expression. Outliers were excluded. (**B)** Expression of MBD2 mRNA in non-neoplastic (*n*=11) and neoplastic (*n*=57) breast tissue specimens. Outliers were excluded.

We used the real-time PCR reaction described above to analyse mRNA expression levels of MeCP2 and MBD2 in non-neoplastic breast tissue (*n*=11) and in breast cancer specimens (*n*=57). The expression levels were standardised using TBP expression. In both groups, we found mRNA expression of MeCP2 and MBD2. We also observed a strong correlation between the expression levels of MeCP2 and MBD2 mRNA in both tissue types (*r*=0.475, *P*⩽0.0001; Figure 1

). Only MeCP2 mRNA expression levels were found to be statistically significantly higher in breast cancer specimens than in non-neoplastic lesions (*P*=0.001; Figure 2A

). No differences in MBD2 expression levels were seen between the two groups (*P*=0.3; Figure 2B). An age difference between patients with non-neoplastic and neoplastic lesions prompted us to use logistic regression analysis: MeCP2 mRNA levels predicted tissue type (non-neoplastic *vs* neoplastic; *P*=0.045) independent of age.

### mRNA expression of MeCP2 and MBD2 in relation to clinicopathologic features in human neoplastic breast tissue

We correlated mRNA expression levels of MeCP2 and MBD2 in tissue specimens of breast cancer patients (*n*=57) with the most important clinicopathological features (grading, histological type of cancer, nodal status, HER2-status, menopausal status, tumour diameter, OR and PR status, overall survival and disease-free survival).

**Figure 3:**
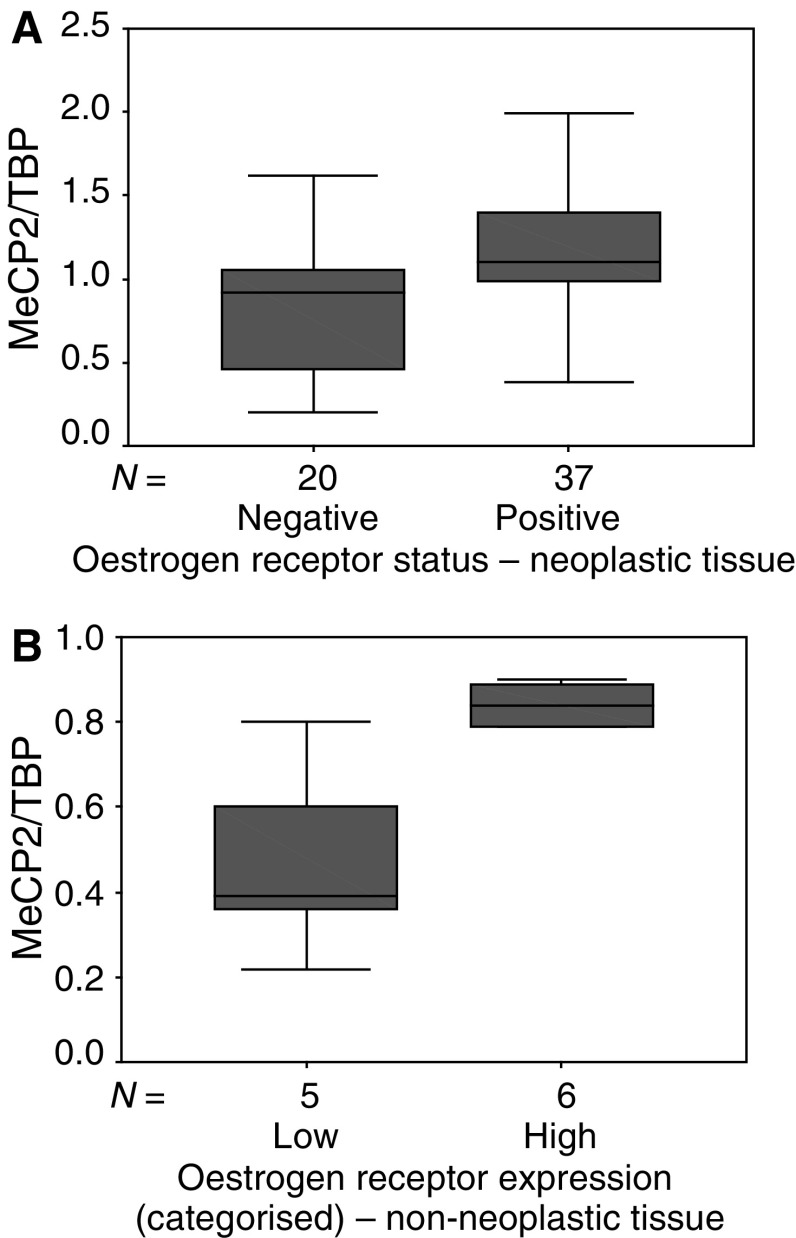
(**A**) Expression of MeCP2 mRNA in OR pos/neg neoplastic breast tissue specimens (*n*=57). Expression levels were analysed using a quantitative real-time PCR method, as described in Materials and Methods. Expression levels were standardised using TBP expression. Outliers were excluded. (**B**) Expression of MeCP2 mRNA in non-neoplastic (*n*=11) breast tissue specimens as a function of OR mRNA expression. Outliers were excluded.

We found a relationship between mRNA expression levels of MeCP2 and OR status: MeCP2 mRNA expression levels were statistically significantly higher in OR-positive as compared to OR-negative breast cancer specimens (*P*=0.005; Figure 3A

). No differences were observed in MBD2 mRNA expression levels.

To study whether the same is true for non-neoplastic tissue, we analysed mRNA expression of OR and PR in the non-neoplastic tissue group (*n*=11). We grouped the samples according to high OR or PR expression. The ‘OR mRNA high’ group demonstrated significantly higher MeCP2 mRNA levels than did the ‘OR mRNA low’ group (*P*=0.017; Figure 3B), whereas MBD2 levels did not differ between these groups (data not shown). Progesterone levels had no impact on MBD2 or MeCP2 expression (data not shown).

**Figure 4:**
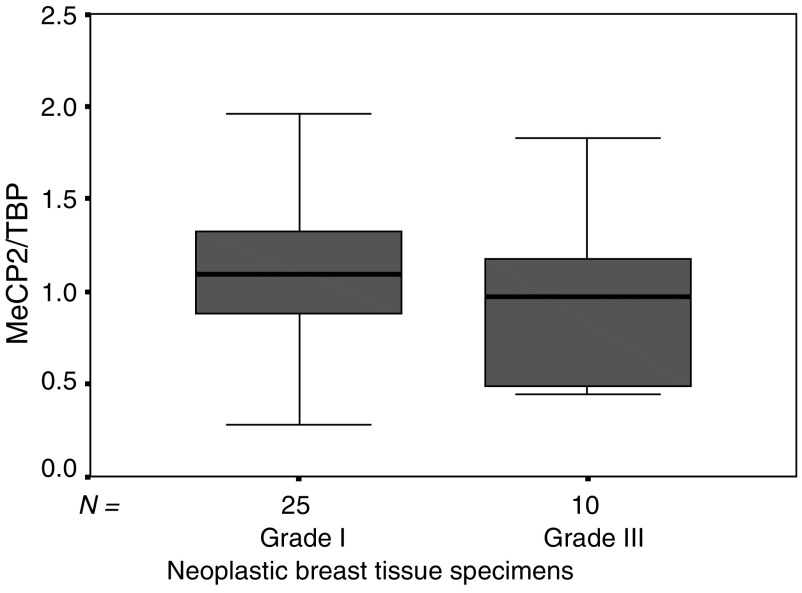
Expression of MeCP2 mRNA in grade I (*n*=25) and grade III (*n*=10) breast cancer lesions. Expression levels were analysed using a quantitative real-time PCR method, as described in Materials and Methods. Expression levels were standardised using TBP expression. Outliers were excluded.

Looking solely at grade I and grade III breast cancer lesions, we observed a visible trend towards lower levels of MeCP2 in grade III lesions as compared to grade I lesions (Figure 4

). This association was not statistically significant, which may be due to the small sample size of the two analysed groups. Investigation of the other clinicopathologic features and mRNA expression of MeCP2 and MBD2 showed no statistically significant differences (data not shown).

### MeCP2 mRNA expression predicts OR mRNA expression in human non-neoplastic and neoplastic breast tissue specimens

A very strong correlation between OR and PR status (identified immunohistochemically and/or biochemically) and the OR and PR mRNA expression in breast cancer specimens measured by RT–PCR was seen (*r*=0.690, *P*⩽0.0001; *r*=0.714, *P*⩽0.0001, respectively).

A relationship between OR and PR is widely known to exist. In our analysis, we found this strong correlation between OR and PR in neoplastic tissue (*r*=0.650, *P*⩽0.0001, respectively), and a statistically nonsignificant trend in non-neoplastic tissue.

To address whether MeCP2 or MBD2 predicts OR mRNA independent of PR, age, interaction of MBD2 and MeCP2 (see Figure 1; expressed by a multiplication of MeCP2 with MBD2 values) and tissue type (non-neoplastic or neoplastic), we used linear regression: MeCP2 independently predicted OR mRNA expression (*P*=0.003). Using PR mRNA expression as an outcome variable, only OR mRNA remains as a statistically significant predictor.

## DISCUSSION

So far only few studies have analysed mRNA expression of MBD2 and MeCP2 in human non-neoplastic and neoplastic tissues (Kanai *et al*, 1999; Müller-Tidow *et al*, 2001; Billard *et al*, 2002). This is a new report focusing solely on the expression behaviour of MBD2 and MeCP2 in non-neoplastic and neoplastic breast tissues.

We were able to demonstrate for the first time a statistically significant correlation in the mRNA expression of MBD2 and MeCP2. Interestingly, we observed only a reduction in MeCP2 mRNA in non-neoplastic tissue as compared to breast cancer tissue. With regard to MBD2 mRNA expression, we did not find different expression levels. We have no explanation for the phenomenon that only MeCP2 is dysregulated, while both MBDs obviously correlate in their expression behaviour. Our findings also contradict the published data, because, as mentioned above, only a reduction in MBD2 has been detected in human neoplasia (Kanai *et al*, 1999; Müller-Tidow *et al*, 2001; Billard *et al*, 2002). There are several reasons that may explain the different findings. First of all, two studies (Kanai *et al*, 1999; Müller-Tidow *et al*, 2001) did not exclusively use breast tissue, as we did in our investigations. As we know from other studies, the expression levels of MeCP2 differ depending on the tissue type analysed (Meehan *et al*, 1992; Tate *et al*, 1996). Therefore, the use of a panel of tumour tissues is more likely to produce a different expression profile than is the use of only one tissue type. For the same reason, a comparison of digestive cancer tissue and breast cancer tissue might well be problematic. A second explanation may be the methodological differences between our study and that of Billard *et al* (2002), who used a competitive RT–PCR method to measure the absolute amount of an mRNA sample, not related to the amount of another control mRNA.

This study also reports for the first time a strong association between OR status and MeCP2 mRNA expression in human breast tissue specimens. So far only Billard *et al* (2002) published a report dealing with MBD2 and MeCP2 mRNA expression in human breast tissue, but they did not find any correlation between OR status and MeCP2 expression level. As mentioned above, the methodological differences and the small sample size used in their study are possible reasons for the different findings.

We found this strong association between high levels of MeCP2 mRNA and OR positivity not only at the protein level of OR (immunohistochemically and/or biochemically determined) but also at the mRNA level of OR (using real-time PCR). As a second step, we aimed to exclude all possible influences on OR expression in order to check whether the expression level of MeCP2 is independently associated with OR status in human breast cancer. We also included the non-neoplastic tissues to address the question of whether this is a general phenomenon in breast tissue or whether it is true only for breast cancer tissue. For this reason, we designed a statistical linear regression model, which included PR mRNA expression and age (both known to have a strong influence on OR mRNA expression), MeCP2, MBD2 mRNA expression and MeCP2 and MBD2 interactions (expressed as the product of both values). Using this model, we observed that the expression level of MeCP2 mRNA is independently associated with the expression level of OR. Using PR mRNA expression as an outcome variable, only OR mRNA remains as a statistically significant predictor. For several reasons, we speculate that MeCP2 – as one of the most important mediators of the epigenetic signal – is regulated by OR and therefore plays a key role in the differentiation processes in human breast tissues:

First of all, it is known that MeCP2 plays a central role in cellular differentiation and development: MeCP2 is not expressed during proliferation or *in vitro* differentiation of the embryonic stem cells, but becomes important at the organ differentiation stage (Li *et al*, 1992; Tate *et al*, 1996). Furthermore, a significant upregulation of MBD2 and MeCP2 mRNA during prenatal development of the human mammary gland has been reported (Billard *et al*, 2002). From these findings concerning the behaviour of MeCP2 during embryonic development, it also seems to be plausible that higher levels of MeCP2 mRNA can be found in better-differentiated human breast cells, indicated by OR positivity.

Recently, an indication of the role of MeCP2 in human development was also provided by linking mutations in the MeCP2 gene to the human neurodevelopmental disorder Rett syndrome (RTT) (Amir *et al*, 1999). Rett syndrome is thought to stem from excessive transcriptional noise due to the failure of MeCP2 to silence genes (Amir and Zoghbi, 2000; Clayton-Smith *et al*, 2000). Other studies also reported a change in the expression level of various genes associated with MeCP2 mutations (Cheadle *et al*, 2000; Traynor *et al*, 2002).

Anti-MeCP2 staining reveals a uniform pattern, suggesting that 5-mC is distributed throughout the genome, and that binding of 10^6^ MeCP2 molecules per cell nucleus may suppress transcriptional noise rather than specific genes (Nan *et al*, 1997).

As we know from preliminary studies in our laboratory with the OR-positive breast cancer cell line MCF-7, OR mRNA is downregulated after treatment with oestrogen. This downregulation of OR mRNA is followed by a downregulation of MeCP2 mRNA, indicating a direct regulation of MeCP2 mRNA via the OR pathway (data not shown).

Another important finding – that fits well into our observations – suggests that a complex formation between DNMT1 and MeCP2 is necessary for maintenance of methylation *in vivo* and was recently reported by Kimura and Shiota (2003).

Overall, we speculate that during differentiation processes in human breast tissues, OR induces the expression of MeCP2, which consequently binds to methylated DNA sequences producing a more compact chromatin state because of interaction with the histone deacetylases and diminishing transcriptional noise. The complex formation between DNMT1 and MeCP2 is also necessary for maintenance of methylation *in vivo*. This also guarantees a transfer of the overall methylation pattern to the daughter cells during mitosis, producing a stable differentiated type of tissue. This hypothesis is also supported by the finding that we detected a visible trend towards lower levels of MeCP2 in grade III lesions as compared to grade I lesions (Figure 4), even though this association is not statistically significant. A decrease in MeCP2/DNMT1 complexes may cause a decrease in DNA methylation and subsequently dedifferentiation of the tumour. Such a connection between DNA hypomethylation in special regions (chromosomes 1 and 16) and tumour dedifferentiation has been shown for breast cancer (Tsuda *et al*, 2002). DNA hypomethylation in general seems to play a central role in carcinogenesis, possibly promoted by chromosomal instability (Eden *et al*, 2003; Gaudet *et al*, 2003).

This study shows for the first time that a strong association between OR status and mRNA expression of the methyl-CpG-binding protein MeCP2, known as a mediator of the epigenetic signal, exists in human breast tissues. Whether MeCP2 is directly involved in the OR pathway or its upregulation is only a general phenomenon of differentiation in breast tissue represented by the expression of OR remains to be further analysed.

## References

[bib1] Amir RE, Van den Veyver IB, Wan M, Tran CQ, Francke U, Zoghbi HY (1999) Rett syndrome is caused by mutations in X-linked MECP2, encoding methyl-CpG-binding protein 2. Nat Genet 23: 185–188 10508514 10.1038/13810

[bib2] Amir RE, Zoghbi HY (2000) Rett syndrome: methyl-CpG-binding protein 2 mutations and phenotype–genotype correlations. Am J Med Genet 97: 147–152 11180222 10.1002/1096-8628(200022)97:2<147::aid-ajmg6>3.0.co;2-o

[bib3] Bieche I, Franc B, Vidaud D, Vidaud M, Lidereau R (2001) Analyses of MYC, ERBB2, and CCND1 genes in benign and malignant thyroid follicular cell tumors by real-time polymerase chain reaction. Thyroid 11: 147–152 11288983 10.1089/105072501300042802

[bib4] Billard LM, Magdinier F, Lenoir GM, Frappart L, Dante R (2002) MeCP2 and MBD2 expression during normal and pathological growth of the human mammary gland. Oncogene 21: 2704–2712 11965543 10.1038/sj.onc.1205357

[bib5] Bird AP, Wolffe AP (1999) Methylation-induced repression – belts, braces, and chromatin. Cell 99: 451–454 10589672 10.1016/s0092-8674(00)81532-9

[bib6] Cheadle JP, Gill H, Fleming N, Maynard J, Kerr A, Leonard H, Krawczak M, Cooper DN, Lynch S, Thomas N, Hughes H, Hulten M, Ravine D, Sampson JR, Clarke A (2000) Long-read sequence analysis of the MECP2 gene in Rett syndrome patients: correlation of disease severity with mutation type and location. Hum Mol Genet 9: 1119–1129 10767337 10.1093/hmg/9.7.1119

[bib7] Chen RZ, Akbarian S, Tudor M, Jaenisch R (2001) Deficiency of methyl-CpG binding protein-2 in CNS neurons results in a Rett-like phenotype in mice. Nat Genet 27: 327–331 11242118 10.1038/85906

[bib8] Clayton-Smith J, Watson P, Ramsden S, Black GC (2000) Somatic mutation in MECP2 as a non-fatal neurodevelopmental disorder in males. Lancet 356: 830–832 11022934 10.1016/s0140-6736(00)02661-1

[bib9] Eden A, Gaudet F, Waghmare A, Jaenisch R (2003) Chromosomal instability and tumors promoted by DNA hypomethylation. Science 300: 455 12702868 10.1126/science.1083557

[bib10] Fishman J, Osborne MP, Telang NT (1995) The role of estrogen in mammary carcinogenesis. Ann N Y Acad Sci 768: 91–100 8526389 10.1111/j.1749-6632.1995.tb12113.x

[bib11] Gaudet F, Hodgson JG, Eden A, Jackson-Grusby L, Dausman J, Gray JW, Leonhardt H, Jaenisch R (2003) Induction of tumors in mice by genomic hypomethylation. Science 300: 489–492 12702876 10.1126/science.1083558

[bib12] Govind AP, Thampan RV (2001) Proteins interacting with the mammalian estrogen receptor: proposal for an integrated model for estrogen receptor mediated regulation of transcription. J Cell Biochem 80: 571–579 11169741 10.1002/1097-4644(20010315)80:4<571::aid-jcb1011>3.0.co;2-h

[bib13] Guy J, Hendrich B, Holmes M, Martin JE, Bird A (2001) A mouse Mecp2-null mutation causes neurological symptoms that mimic Rett syndrome. Nat Genet 27: 322–326 11242117 10.1038/85899

[bib14] Hendrich B, Bird A (1998) Identification and characterization of a family of mammalian methyl-CpG binding proteins. Mol Cell Biol 18: 6538–6547 9774669 10.1128/mcb.18.11.6538 PMC109239

[bib15] Hendrich B, Guy J, Ramsahoye B, Wilson VA, Bird A (2001) Closely related proteins MBD2 and MBD3 play distinctive but interacting roles in mouse development. Genes Dev 15: 710–723 11274056 10.1101/gad.194101 PMC312657

[bib16] Iwao K, Miyoshi Y, Egawa C, Ikeda N, Tsukamoto F, Noguchi S (2000) Quantitative analysis of estrogen receptor-alpha and -beta messenger RNA expression in breast carcinoma by real-time polymerase chain reaction. Cancer 89: 1732–1738 11042568 10.1002/1097-0142(20001015)89:8<1732::AID-CNCR13>3.0.CO;2-2

[bib17] Jones PA, Baylin SB (2002) The fundamental role of epigenetic events in cancer. Nat Rev Genet 3: 415–428 12042769 10.1038/nrg816

[bib18] Kanai Y, Ushijima S, Nakanishi Y, Hirohashi S (1999) Reduced mRNA expression of the DNA demethylase, MBD2, in human colorectal and stomach cancers. Biochem Biophys Res Commun 264: 962–966 10544038 10.1006/bbrc.1999.1613

[bib19] Kimura H, Shiota K (2003) Methyl-CpG binding protein, MeCP2, is a target molecule for maintenance DNA methyltransferase, Dnmt1. J Biol Chem 278: 4806–4812 12473678 10.1074/jbc.M209923200

[bib20] Laird PW (2003) Early detection: the power and the promise of DNA methylation markers. Nat Rev Cancer 3: 253–266 12671664 10.1038/nrc1045

[bib21] Lewis JD, Meehan RR, Henzel WJ, Maurer-Fogy I, Jeppesen P, Klein F, Bird A (1992) Purification, sequence, and cellular localization of a novel chromosomal protein that binds to methylated DNA. Cell 69: 905–914 1606614 10.1016/0092-8674(92)90610-o

[bib22] Li E, Bestor TH, Jaenisch R (1992) Targeted mutation of the DNA methyltransferase gene results in embryonic lethality. Cell 69: 915–926 1606615 10.1016/0092-8674(92)90611-f

[bib23] Magdinier F, Wolffe AP (2001) Selective association of the methyl-CpG binding protein MBD2 with the silent p14/p16 locus in human neoplasia. Proc Natl Acad Sci USA 98: 4990–4995 11309512 10.1073/pnas.101617298 PMC33151

[bib24] McGuire WL (1978) Hormone receptors: their role in predicting prognosis and response to endocrine therapy. Semin Oncol 5: 428–433 734443

[bib25] Meehan RR, Lewis JD, Bird AP (1992) Characterization of MeCP2, a vertebrate DNA binding protein with affinity for methylated DNA. Nucleic Acids Res 20: 5085–5092 1408825 10.1093/nar/20.19.5085 PMC334288

[bib26] Meehan RR, Lewis JD, McKay S, Kleiner EL, Bird AP (1989) Identification of a mammalian protein that binds specifically to DNA containing methylated CpGs. Cell 58: 499–507 2758464 10.1016/0092-8674(89)90430-3

[bib27] Müller C, Readhead C, Diederichs S, Idos G, Yang R, Tidow N, Serve H, Berdel WE, Koeffler HP (2000) Methylation of the cyclin A1 promoter correlates with gene silencing in somatic cell lines, while tissue-specific expression of cyclin A1 is methylation independent. Mol Cell Biol 20: 3316–3329 10757815 10.1128/mcb.20.9.3316-3329.2000 PMC85625

[bib28] Müller HM, Widschwendter M (2003) Methylated DNA as a possible screening marker for neoplastic disease in several body fluids. Expert Rev Mol Diagn 3: 443–458 12877384 10.1586/14737159.3.4.443

[bib29] Müller-Tidow C, Kugler K, Diederichs S, Klumpen S, Moller M, Vogt U, Metzger R, Schneider PM, Berdel WE, Serve H (2001) Loss of expression of HDAC-recruiting methyl-CpG-binding domain proteins in human cancer. Br J Cancer 85: 1168–1174 11710831 10.1054/bjoc.2001.2041 PMC2375156

[bib30] Nan X, Campoy FJ, Bird A (1997) MeCP2 is a transcriptional repressor with abundant binding sites in genomic chromatin. Cell 88: 471–481 9038338 10.1016/s0092-8674(00)81887-5

[bib31] Nan X, Meehan RR, Bird A (1993) Dissection of the methyl-CpG binding domain from the chromosomal protein MeCP2. Nucleic Acids Res 21: 4886–4892 8177735 10.1093/nar/21.21.4886 PMC311401

[bib32] Tate P, Skarnes W, Bird A (1996) The methyl-CpG binding protein MeCP2 is essential for embryonic development in the mouse. Nat Genet 12: 205–208 8563762 10.1038/ng0296-205

[bib33] Traynor J, Agarwal P, Lazzeroni L, Francke U (2002) Gene expression patterns vary in clonal cell cultures from Rett syndrome females with eight different MECP2 mutations. BMC Med Genet 3: 12 12418965 10.1186/1471-2350-3-12 PMC137585

[bib34] Tsuda H, Takarabe T, Kanai Y, Fukutomi T, Hirohashi S (2002) Correlation of DNA hypomethylation at pericentromeric heterochromatin regions of chromosomes 16 and 1 with histological features and chromosomal abnormalities of human breast carcinomas. Am J Pathol 161: 859–866 12213714 10.1016/S0002-9440(10)64246-0 PMC3277345

[bib35] Wade PA (2001) Methyl CpG binding proteins: coupling chromatin architecture to gene regulation. Oncogene 20: 3166–3173 11420733 10.1038/sj.onc.1204340

[bib36] Widschwendter M, Berger J, Hermann M, Müller HM, Amberger A, Zeschnigk M, Widschwendter A, Abendstein B, Zeimet AG, Daxenbichler G, Marth C (2000) Methylation and silencing of the retinoic acid receptor-beta2 gene in breast cancer. J Natl Cancer Inst 92: 826–832 10814678 10.1093/jnci/92.10.826

